# Branched-Chain Amino Acids and Arginine Improve Performance in Two Consecutive Days of Simulated Handball Games in Male and Female Athletes: A Randomized Trial

**DOI:** 10.1371/journal.pone.0121866

**Published:** 2015-03-24

**Authors:** Chen-Kang Chang, Kun-Ming Chang Chien, Jung-Hsien Chang, Mei-Hsuan Huang, Ya-Chuan Liang, Tsung-Han Liu

**Affiliations:** 1 Sport Science Research Center, National Taiwan University of Sport, Taichung, Taiwan; 2 Department of Ball Sport, National Taiwan University of Sport, Taichung, Taiwan; 3 Office of Physical Education, National Taiwan University of Sport, Taichung, Taiwan; 4 Institute of Sport Performance, National Taiwan University of Sport, Taichung, Taiwan; 5 Graduate Institute of Sports Training, University of Taipei, Taipei, Taiwan; University of the Balearic Islands, SPAIN

## Abstract

The central nervous system plays a crucial role in the development of physical fatigue. The purpose of this study is to investigate the effect of combined supplementation of branched-chain amino acids (BCAA) and arginine on intermittent sprint performance in simulated handball games on 2 consecutive days. Methods: Fifteen male and seven female handball players consumed 0.17 g/kg BCAA and 0.04 g/kg arginine together (AA trial), or placebo (PB trial) before exercise. Each trial contained two 60-min simulated handball games on consecutive days. The game was consisted of 30 identical 2-min blocks and a 20 m all-out sprint was performed at the end of each block. The performance, measured by percentage changes of sprint time between day 1 and 2, was significantly better in the AA trial (first half: AA trial: -1.34±0.60%, PB trial: -0.21±0.69%; second half: AA trial: -1.68±0.58%, PB trial: 0.49±0.42%). The average ratings of perceive exertion throughout the 2-day trial was significantly lower in the AA trial (14.2±0.3) than the PB trial (15.1±0.4). Concurrently, post-exercise tryptophan/BCAA ratio on both days in the AA trial was significantly lower than the baseline. This study showed that BCAA and arginine supplementation could improve performance in intermittent sprints on the second consecutive day of simulated handball games in well-trained athletes by potentially alleviating central fatigue.

## Introduction

The central nervous system plays a crucial role in the development of physical fatigue. One of the proposed mechanisms that contribute to central fatigue is the increase in blood concentration of free tryptophan and hence the neurotransmitter serotonin (5-hydroxytryptamine) in the brain during exercise [1]. The elevated plasma non-esterified fatty acid (NEFA) concentration during sustained exercise could increase the plasma level of free tryptophan because they compete for the same binding site in albumin [2]. The transport of tryptophan across the blood-brain barrier is the rate-limiting step in the cerebral synthesis of serotonin [3]. Serotonin in the brain is involved in the control of arousal, sleepiness and mood. Therefore, it has been suggested that the activation of the brain serotonin system would lead to the development of fatigue during exercise [4]. To support this hypothesis, the time to exhaustion in endurance exercise was significantly decreased by the administration of serotonin agonists, while it was increased by serotonin antagonists or inhibitors of serotonin synthesis in humans and rats [5–8].

Branched-chain amino acids (BCAA) have been proposed to alleviate central fatigue due to their ability to compete with tryptophan in crossing blood-brain barrier through the same L-system transporter [3]. Therefore, the decreased plasma tryptophan/BCAA ratio after BCAA supplementation would reduce the cerebral uptake of tryptophan and, subsequently, serotonin synthesis [1]. Indeed, animal studies have shown that BCAA could increase running time to exhaustion, which is accompanied by the reduced plasma free tryptophan/BCAA ratio and exercise-induced cerebral synthesis and release of serotonin [9–12]. However, most human studies have failed to find improvement in endurance performance [5,13–15]. Although the indicators of central fatigue, such as perceived ratings of fatigue and cognitive functions, were improved after BCAA supplementation [13,15].

One possible explanation for the lack of effect of BCAA supplementation on exercise performance in humans is the accompanied further increase in NH_3_ production, resulted from the elevated BCAA oxidation [5,14,16–18]. It would result in elevated cerebral uptake and accumulation of NH_3_ [19], leading to central fatigue by alterations of cerebral energy metabolism, neurotransmission, and signaling pathways within the neuron [20]. Therefore, the potential benefit of BCAA on central fatigue may be offset by the simultaneous increase in NH_3_. Arginine has been suggested to be able to reduce exercise-related accumulations of NH_3_ by increasing urea cycle [21,22] and vasodilation [23]. Thus, the current study combined BCAA and arginine to alleviate central fatigue by decreasing tryptophan/BCAA ratio while preventing hyperammonemia.

Most studies investigating the effect of BCAA on central fatigue and performance focused on a single bout of endurance exercise. One form of exercise that has received little attention is the intermittent sprint, an important activity pattern in many team sports, such as handball, basketball, and soccer. A recent study has shown the decreases in cognitive function and reactive motor skills after exhaustive intermittent exercise in athletes, indicating the presence of central fatigue [24]. The fatigue factors may become more prominent during national and international tournaments in these sports because the competitions are usually arranged on consecutive days. However, the role of central fatigue in consecutive days of intermittent exercise has not been examined. Therefore, the purpose of this study is to investigate the combined supplementation of BCAA and arginine on performance in intermittent sprints in simulated handball games on 2 consecutive days in well-trained athletes.

## Materials and Methods

### Subjects

The subjects were 15 male and 7 female well-trained handball players recruited from National Taiwan University of Sport, Taichung, Taiwan. All subjects have competed at the national or international level. The characteristics of subjects are shown in Table 1. The subjects were free of known cardiovascular disease risks and musculoskeletal injuries. The subjects had not taken any protein supplement for at least 3 months prior to the study. The regular training schedule and diet habits were maintained during the study period, except on the day before each trial when all training was avoided and standardized meals were provided. All subjects gave their written informed consent after the experimental procedure and potential risks were explained. The study protocol was approved by the Human Subject Committee of National Taiwan University of Sport.

**Table 1:** Characteristics of male and female subjects, mean±SD.

Parameters	Male (n = 15)	Female (n = 7)
**Age (years)**	21.1±1.0	20.3±0.5
**Height (m)**	1.80±0.07	1.61±0.04
**Weight (kg)**	78.3±11.7	53.9±5.0
**Estimated VO**_**2max**_ **(ml/kg/min)**	52.3±4.6	48.6±2.9
**Training experience (years)**	11.1±2.0	10.0±0.6

### Study design

**Fig 1:**
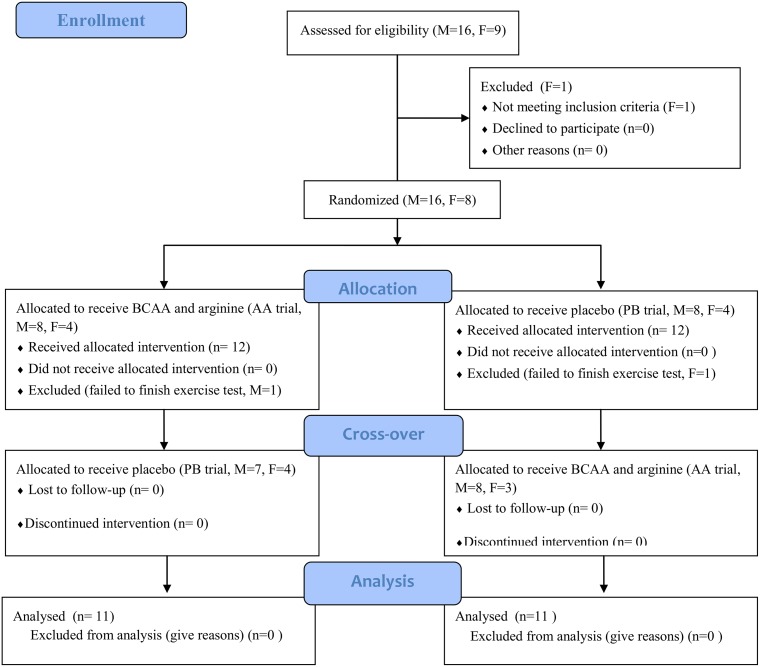
Study design and flow of participants through the study.

This study used a double-blind, randomized cross-over design (Fig. 1). Each subject completed AA and placebo (PB) trials in a random order, separated by a wash-out period of 7–14 days. The randomization was stratified on gender. Each trial contained 2 consecutive days with 1 simulated handball game on each day. The start time of the simulated game was the same in both trials for the same subject to ensure that all parameters were collected at the same time points. The same food was provided in the lunch and dinner on the day before, and during the 2-day trial period. The lunch and dinner were meal boxes purchased from a local restaurant, providing approximately 1560 kcal in total, with 45.7% energy from carbohydrate, 31.2% from fat, and 22.1% from protein. The diet analysis was performed by a dietitian using Taiwanese food exchange table [25]. The breakfast on the days of simulated games included white bread 1.2 g/kg, jam 0.1 g/kg, butter 0.l g/kg, and soybean milk 5 ml/kg (6.2 kcal/kg, containing carbohydrate 1.0 g/kg, protein 0.24 g/kg, and fat 0.14 g/kg).

### Measurement of cardiopulmonary function

Approximately 1 week prior to the first trial, the cardiopulmonary function of the subjects was measured with a multistage 20 m shuttle run in an indoor gym with wooden floor. This test has been shown to provide a valid and reliable estimation of VO_2max_ for team sport players [26]. The pace was set in a pre-recorded MP3 file, starting at 8.5 km/hr and increasing by 0.5 km/hr every 2 min. The test was stopped if the subject failed to finish 20 m within the required time for 2 consecutive runs. VO_2max_ was estimated according to the number of stages completed [26].

### Experimental procedure

On the days of the trials, the subjects reported to the laboratory in the early morning after an overnight fast, then consumed the standardized breakfast. After the breakfast, the subjects in the AA trial ingested 0.17 g/kg BCAA (leucine: isoleucine: valine = 2:1:1, powdered form, Optimum Nutrition, Inc, Aurora, IL, USA) and 0.04 g/kg arginine (in capsule, General Nutrition Corporation, Pittsburgh, PA, USA). BCAA powder was dissolved in 250 ml grape juice to mask the taste. In the PB trial, the subjects ingested starch powder and capsules containing starch as the placebo. The starch power, at the same amount as the BCAA powder in the AA trial, was also dissolved in 250 ml of grape juice. The number of capsule ingested in the PB trial was the same as that in the AA trial. Our preliminary study has shown that the plasma BCAA and arginine concentrations would peak after 1 hr of ingestion (data not shown). Therefore, the supplementations were consumed 1 hr before the exercise in this study.

The simulated handball games started 60 min after the subjects finished the breakfast. The subjects were allowed to drink water ad libitum in the first trial, while the timing and amount of consumption were recorded. The timing and amount of water consumption were repeated in the second trial. The average water consumption on day 1 and 2 was 467±44 and 503±49 ml, respectively.

### Simulated handball game and performance measurement

The simulated game was designed in conjunction with the coaches of Taiwanese national team to mimic the activity patterns of real handball competitions. Each simulated game contained two 30-min halves with a 10-min rest in between. The game was consisted of 30 identical 2-min blocks with each block containing (1) 3 m side-steps x 3 in 10 sec, (2) 20 m run in 5 sec, (3) 5 passes and 1 jumping shot in 10 sec, (4) 20 m jogging in 10 sec, (5) single-leg cross-hops x 3 in 10 sec, (6) 20 m run in 5 sec, (7) 8 passes in 10 sec, (8) 20 m jog in 15 sec, (9) 20 m all-out sprint. The time of each 20 m all-out sprint was recorded using photocells (Powertimers 300-series, Newtest, Oulu, Finland). Vocal encouragement was provided by the research personnel throughout the game. The average time of sprints in the first and second halves was used as the indicator of exercise performance. The percentage changes of sprint time between day 1 and day 2 on the same trial were calculated as followed.

Percentage changes of sprint time=(sprint time on day2– sprint time on day1)×100/sprint time on day1

The ratings of perceived exertion (RPE) were recorded immediately after each half of the simulated game using Borg’s 20-point scale [27]. Heart rate was recorded throughout the simulated games by telemetry (Heart rate monitor, Polar, Lake Success, NY, USA)

### Blood sample collection

Venous blood samples were collected before breakfast and immediately after the simulated handball games on both days. Ten milliliters of blood sample was collected into a tube containing EDTA as anticoagulant. The blood samples were centrifuged at 1500 x g (Eppendorf 5810, Hamburg, Germany) to extract plasma. The aliquoted plasma samples were stored at -70°C until further analysis.

### Measurement of blood biochemical parameters

Hemoglobin concentration and hematocrit in whole blood was measured by a hematology analyzer (KX-21N, Sysmex Corporation, Kobe, Japan) to correct for the change in plasma volume [28]. Plasma BCAA concentration was measured enzymatically according to manufacturer’s recommendation (Biovision, Milpitas, CA, USA). The absorbance at 450 nm was measured with a microplate spectrophotometer (Benchmark Plus, Bio-Rad, Hercules, CA, USA). Plasma free tryptophan concentration was analyzed with a fluorescence assay according to manufacturer’s recommendation (Bridge-It, Mediomics, St. Louis, MO, USA). The fluorescence at excitation 485 nm and emission 665 nm was read by a microplate fluorescence reader (Plate Chameleon, Hidex, Turku, Finland). Plasma concentrations of NH_3_, glycerol, NEFA, and lactate were measured with an automatic analyzer (Hitachi 7020, Tokyo, Japan) using commercial kits (Randox, Antrim, UK). The plasma concentrations of all parameters were corrected for the changes in plasma volume before being analyzed statistically.

### Statistical analysis

All values were expressed as mean±SEM unless specified. The variables were initially analyzed by 3-way (trial x time x gender) analysis of variance (ANOVA) with repeated measurements. The trial factor represents AA and PB trial. The time factor represents the 4 time points in each trial, i.e. day 1 pre-exercise, day 1 post-exercise, day 2 pre-exercise, and day 2 post-exercise. The data of male and female subjects were later pooled together because the gender effect was found insignificant. Therefore, the results were analyzed by 2-way (trial x time) ANOVA with repeated measurements. If the main effect was significant, the post hoc Bonferroni analysis was used to identify the difference. A p-value less than. 05 was considered statistically significant.

## Results and Discussion

**Fig 2:**
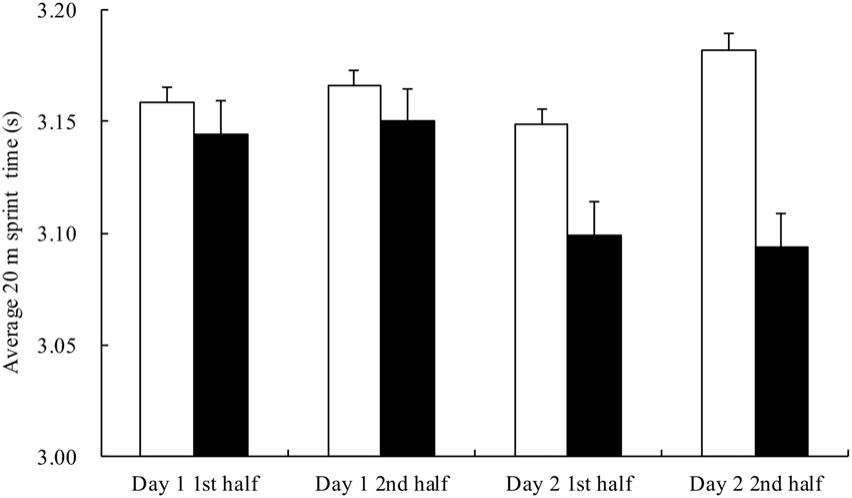
Average 20 m sprint time in the simulated handball games on day 1 and day 2 in the placebo (□) and AA (■) trials. Main effects: trial: NS; time: p = .048; interaction: p = .040

**Fig 3:**
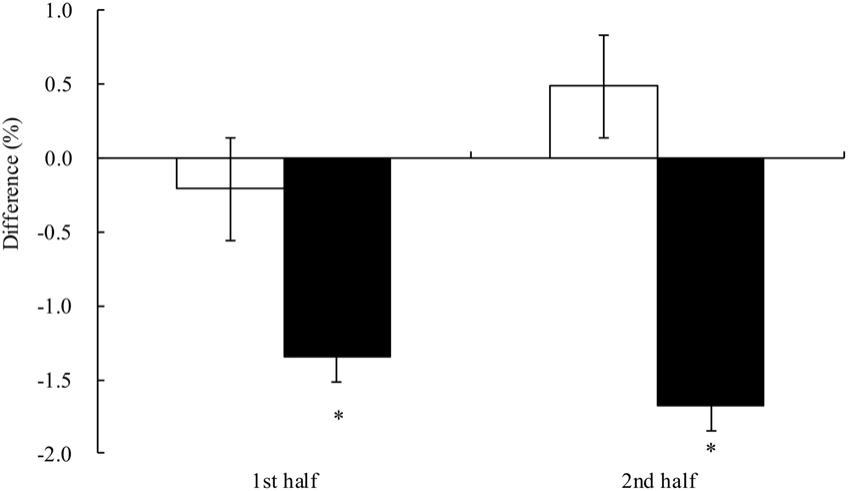
Percentage changes of sprint time in the simulated handball game between day 1 and day 2 in the placebo (□) and AA (■) trials. Main effects: trial: p = .042; time: p = .546; interaction: p = .239. *p<.001 compared to the placebo trial.

The average 20 m sprint time on day 1 and day 2 in both trials is shown in Fig. 2. There were significant time and trial x time effects, but post hoc analysis did not find any significant difference. In order to investigate the performance on day 2, compared to day 1, the differences in sprint time between the 2 days in the same trial were analyzed (Fig. 3). On day 2 in the AA trial, the sprint performance improved by 1.34±0.60% and 1.68±0.58% in the first and second half, respectively, from the previous day. The improvements were significantly better than those in the PB trial (p = .001 in first half; p<.001 in second half).

**Fig 4:**
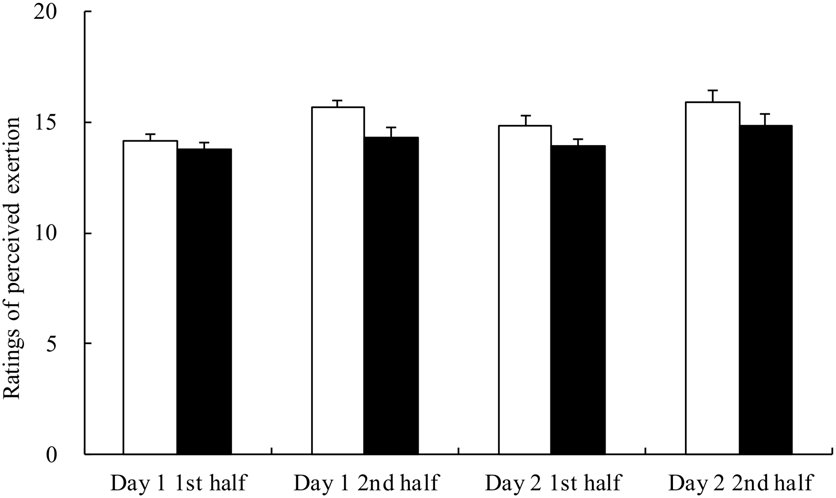
Ratings for perceived exertion after the first and second half of the simulated handball game on day 1 and day 2 in the placebo (□) and AA (■) trials. Main effects: trial: p = .026; time: p<.001; interaction: p<.001.

The RPE after the first and second half of the simulated games in both trials are depicted in Fig. 4. There were significant trial and time effects, but post hoc analysis did not find any significant difference. In further analysis, the average RPE throughout the 2-day trial was indeed lower in the AA trial (14.2±0.3) than PB trial (15.1±0.4, p = .005). The average heart rates during the simulated games on day 1 and 2 were 143–159 bpm with no difference between the trials.

**Fig 5:**
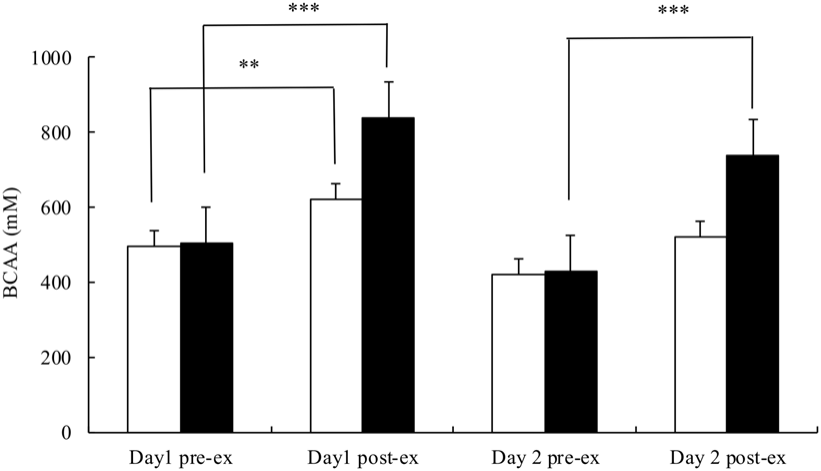
Plasma concentrations of branched-chain amino acids before and after the simulated handball game on day 1 and day 2 in the placebo (□) and AA (■) trials. Main effects: trial: p<.001; time: p<.001; interaction: p<.001. **p<.01; ***p<0.01.

**Fig 6:**
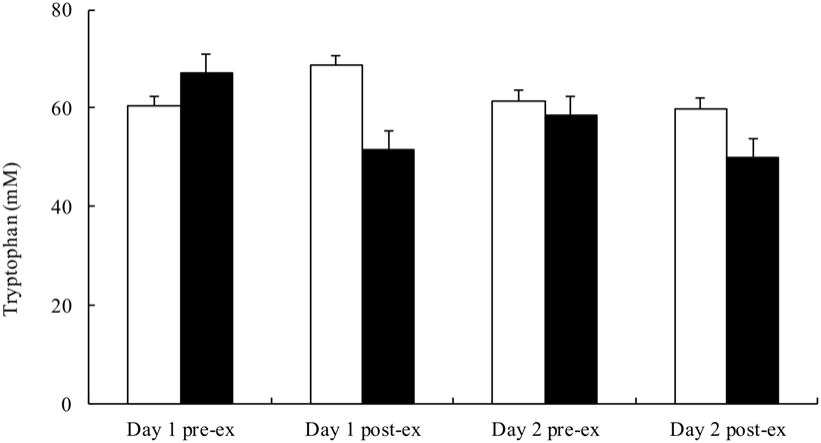
Plasma concentrations of tryptophan before and after the simulated handball game on day 1 and day 2 in the placebo (□) and AA (■) trials. Main effects: trial: p = .124; time: p = .021; interaction: p<.001.

**Fig 7:**
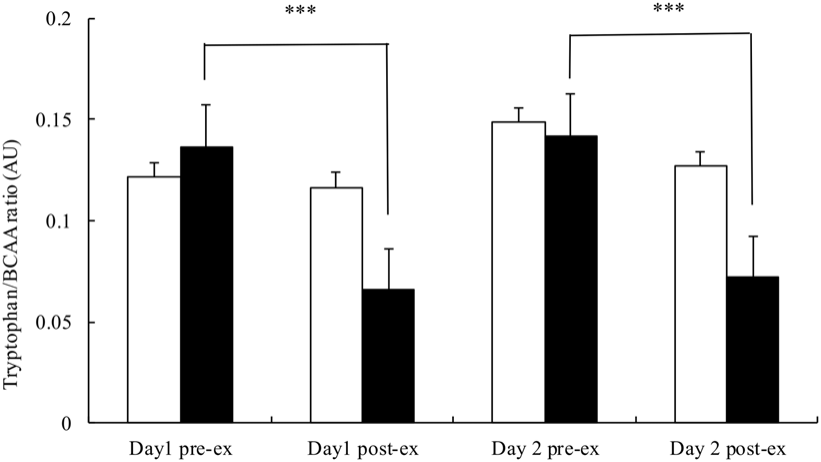
Plasma branched-chain amino acids to tryptophan ratio before and after the simulated handball game on day 1 and day 2 in the placebo (□) and AA (■) trials. Main effects: trial: p = .026; time: p<.001; interaction: p<.001. ***p<0.01.

Post-exercise plasma BCAA concentration was significantly elevated on both days in the AA trial and day 1 in the PB trial (Fig. 5), while tryptophan concentration were similar between the 2 trials (Fig. 6). Consequently, plasma tryptophan to BCAA ratio was significantly lower after exercise on both days (p<.001) in the AA trial, while it remained unchanged in the PB trial (Fig. 7).

Plasma NH_3_ concentrations were significantly elevated after exercise on both trials. In addition, post-exercise NH_3_ concentration was significantly higher in the AA trial than the PB trial on both days (Table 2). Plasma concentrations of lactate, glycerol, and NEFA were all significantly increased after exercise. However, there was no significant difference between the 2 trials at the same time point (Table 2).

**Table 2:** Plasma concentrations of biochemical parameters before and after exercise in the PB and AA trials, mean ± SD.

		Day 1		Day 2	
	Trial^a^	Pre-exercise	Post-exercise	Pre-exercise	Post-exercise
NH_3_ (μM)	PB	44.32±8.56	134.02±21.65*	42.00±7.66	123.87±23.93*
	AA	43.68±8.30	161.82±33.85* ^,^ †	47.86±7.52	152.44±32.56* ^,^ †
NEFA^b^ (mM)	PB	0.26±0.13	0.47±0.25*	0.34±0.18	0.55±0.24*
	AA	0.25±0.12	0.35±0.12*	0.38±0.16	0.47±0.16*
Glycerol (μM)	PB	19.14±16.32	85.6±70.04*	25.82±21.36	111.27±78.15*
	AA	18.77±17.06	60.15±31.63*	25.70±21.90	85.06±31.97*
Lactate (mM)	PB	3.48±1.70	6.04±2.35*	2.69±1.22	4.37±1.92*
	AA	3.56±1.57	6.02±1.87*	2.91±1.32	4.29±1.50*

^a^PB: placebo; AA: BCAA+arginine.

^b^NEFA: non-esterified fatty acid.

*Significantly different from pre-exercise at the same day in the same trial (p<.05).

†Significantly different from the PB trial on the same day (p<.05).

The aim of this study is to investigate the effect of BCAA and arginine supplementation on the performance in repeated sprints on 2 consecutive days. To our knowledge, this is the first study that showed the combination of BCAA and arginine could induce a small but significant improvement in performance in intermittent sprints on the second consecutive day in well-trained subjects. The improvement may result from the reduced central fatigue as indicated by lower RPE in the AA trial. However, the central fatigue may be mediated by factors in addition to the reduced serotonin synthesis.

The development of fatigue on day 2 was evident in the PB trial. The sprint time was significantly increased by 1.01±1.49% on the second half, compared to the first half on day 2. On the other hand, in the AA trial the average sprint time on the second half was similar to that in the first half on day 2. In further analysis, the difference between the sprint time on day 1 and day 2 were significantly decreased in the AA trial, compared to the PL trial, indicating an improvement in sprint performance on day 2 in the AA trial.

The average RPE throughout the 2-day trial was indeed significantly lower in the AA trial (14.2±0.3) than the PB trial (15.1±0.4, p = .005). Concurrently, post-exercise tryptophan/BCAA ratio on both days in the AA trial was significantly lower than the baseline level. Nevertheless, post-exercise tryptophan concentration and tryptophan/BCAA ratio remained unchanged in the PB trial, indicating no change in the cerebral serotonin production. It is plausible that the lower tryptophan/BCAA ratio in the AA trial would further reduce the cerebral serotonin synthesis, resulting in lower RPE and better performance.

The central fatigue theory proposed by Blomstrand et al. (1988) has been supported in animal studies. The administration of BCAA or L-system transporter inhibitor significantly increased exercise time to exhaustion in rats by decreasing synaptosomal tryptophan and serotonin synthesis [12,29]. On the other hand, most human studies failed to find ergogenic effect of BCAA in a single bout of intermittent [24] or endurance exercise [1,5,13–15,30,31], despite that some researchers reported reductions in central fatigue. In the present study, significant reductions in tryptophan/BCAA ratio after exercise were found on both days in the AA trial. However, the improvement in sprint performance was only found on day 2. It is possible that the negative effect of central fatigue on physical performance only becomes evident under more physiologically and psychologically stressful conditions after the accumulation of fatigue from the previous day. It could also at least partially explain the disconnection between central fatigue and physical performance in studies using a single bout of exercise.

Unexpectedly, the subjects in both trials did not show an increase in total tryptophan concentration after exercise. As the result, total tryptophan/BCAA ratio remained unchanged after exercise in the PL trial. Although free tryptophan concentrations were not measured in this study, it is reasonable to assume that they were increased after exercise in both trials because the elevated plasma free fatty acid would compete with tryptophan for the same binding site in albumin [2,32]. Therefore, post-exercise free tryptophan/BCAA ratio would be higher than the baseline level in the PL trial. The higher free tryptophan/BCAA ratio would lead to increased serotonin production and central fatigue. In the AA trial the elevated plasma BCAA level would be sufficient to offset the increased free tryptophan concentration, resulting in lower post-exercise free tryptophan/BCAA ratio. Another possibility is that central fatigue in intermittent exercise is mediated by factors in addition to tryptophan/BCAA ratio. Brain function is extremely complicated and involves numerous neurotransmitters. It is very likely that more than one neurotransmitter is responsible for central fatigue. The serotonin functions may be regulated by the interactions with other neurotransmitters such as catecholamines and γ-aminobutyric acid [33]. The hypothesis that serotonin/dopamine ratio, rather than serotonin alone, contributes to central fatigue has also been proposed [34].

The AA trial showed significantly higher post-exercise NH_3_ concentration on both days than that in the PB trial. This is similar to the previous studies using BCAA supplement [14,35]. The arginine supplementation failed to prevent the additional NH_3_ production, possibly from BCAA oxidation, during the simulated games. It is possible that the large amount of NH_3_ production during intermittent sprints may exceed the effect of arginine on urea cycle [36]. In addition, our well-trained subjects may already have high levels of NO production [37]. Therefore, the supplementation of arginine did not provide additional effect on vasodilation and removal of NH_3_. An addition of BCAA-only trial in the future studies would further clarify the role of arginine when co-ingested with BCAA.

The unique experimental protocol in this study, a simulated game on 2 consecutive days, tried to mimic the actual tournament situations. It allows the investigation of BCAA and arginine effects beyond a single bout of exercise that was used in all previous studies. The activity pattern in the simulated game in this study was similar to real professional competitions. A time-motion analysis study showed that professional male handball players performed 22.0±10.0 sprints with distance of 18.0±6.91 m during a match [38]. Similarly, the simulated game in this study is consisted of 30 sprints of 20 m. The average heart rates during the simulated games (143–159 bpm) also resembled those recorded in real competitions (157±18 bpm) [38]. Therefore, it is reasonable to assume that the simulated game used in this study could reflect the physiological demands of high-level handball competitions.

In the AA trial, the sprint performance was slightly improved on day 2, compared to day 1. During the simulated game on day 2 in both trials, higher portions of energy were derived from fat, indicated by the higher post-exercise plasma glycerol and NEFA concentrations. It has been shown that BCAA could increase lipid oxidation and the time to exhaustion in glycogen-depleted subjects [39]. Therefore, it is possible that BCAA could improve the performance on day 2 by promoting lipid oxidation, in addition to relieving central fatigue. This hypothesis warrants further investigation.

## Conclusions

This study suggested that supplementation of BCAA and arginine could improve the performance in intermittent sprint on the second consecutive days of simulated games, possibly by alleviating central fatigue. The suppressed serotonin production, resulted from decreased tryptophan/BCAA ratio may play a role in reducing central fatigue. The results of this study have significant practical applications because the athletes of team sports frequently compete on consecutive days in tournaments. The effect of BCAA and arginine on other neurotransmitters such as dopamine and catecholamines that may be involved in central fatigue need to be further examined. In addition, future studies could add a BCAA-only trial to further clarify the role of arginine on NH_3_ removal and central fatigue.

## Supporting Information

S1 Checklist CONSORT checklist information. (DOC)

S1 Table Dataset of the results. (XLSX)
